# *Escherichia coli *infection induces distinct local and systemic transcriptome responses in the mammary gland

**DOI:** 10.1186/1471-2164-11-138

**Published:** 2010-02-25

**Authors:** Simone Mitterhuemer, Wolfram Petzl, Stefan Krebs, Daniel Mehne, Andrea Klanner, Eckhard Wolf, Holm Zerbe, Helmut Blum

**Affiliations:** 1 Laboratory for Functional Genome Analysis (LAFUGA), Gene Center, LMU Munich, Feodor-Lynen-Str 25, 81377 Munich, Germany; 2 Clinic for Ruminants, Center for Clinical Veterinary Medicine, LMU Munich, Sonnenstr 16, 85764 Oberschleißheim, Germany; 3 Chair for Molecular Animal Breeding and Biotechnology, Gene Center, LMU Munich, Feodor-Lynen-Str 25, 81377 Munich, Germany

## Abstract

**Background:**

Coliform bacteria are the most common etiologic agents in severe mastitis of cows. *Escherichia coli *infections are mostly restricted to a single udder quarter whereas neighboring quarters stay clinically inapparent, implicating the presence of a systemic defense reaction. To address its underlying mechanism, we performed a transcriptome study of mammary tissue from udder quarters inoculated with *E. coli *(6 h and 24 h post infection), from neighboring quarters of the same animals, and from untreated control animals.

**Results:**

After 6 h 13 probe sets of differentially expressed genes (DEG) were detected in infected quarters versus control animals. Eighteen hours later 2154 and 476 DEG were found in infected and in neighboring quarters vs. control animals. Cluster analysis revealed DEG found only in infected quarters (local response) and DEG detected in both infected and neighboring quarters (systemic response). The first group includes genes mainly involved in immune response and inflammation, while the systemic reaction comprises antigen processing and presentation, cytokines, protein degradation and apoptosis. Enhanced expression of antimicrobial genes (*S100A8*, *S100A9*, *S100A12*, *CXCL2*, *GNLY*), acute phase genes (*LBP*, *SAA3*, *CP*, *BF, C6*, *C4BPA*, *IF*), and indicators of oxidative stress (*GPX3*, *MT1A*, *MT2A*, *SOD2*) point to an active defense reaction in infected and neighboring healthy quarters. Its early onset is indicated by increased transcription of *NFIL3 *at 6 h. NFIL3 is a predicted regulator of many genes of the systemic response at 24 h. The significance of our transcriptome study was evidenced by some recent findings with candidate gene based approaches.

**Conclusions:**

The discovery and holistic analysis of an extensive systemic reaction in the mammary gland significantly expands the knowledge of host-pathogen interactions in mastitis which may be relevant for the development of novel therapies and for genetic selection towards mastitis resistance.

## Background

Udder infections cause considerable economic losses to the dairy industry [1] and often lead to culling of affected animals [2]. Due to the economic importance of mastitis and the health risk for consumers, large efforts have been made to identify factors involved in the susceptibility of dairy cows to infections of the mammary gland [3,4] which occur most frequently at parturition [5]. In that period, infections with *E. coli *often cause severe clinical symptoms [6,7] accompanied by reduction in milk yield, altered milk composition and extensive damage of mammary tissue [8]. *E. coli *infection predominantly affects a single udder quarter [9]. The other quarters are clinically asymptomatic and are considered to be healthy. Nevertheless, there are indications that neighboring apparently healthy udder quarters are infected without showing clinical changes. Epidemiological studies revealed, that the identical environmental strain of *E. coli *can cause recurrent mastitis, but in different udder quarters relative to the one infected first [10]. This phenomenon was attributed to initial infection of multiple quarters, each at a different level and with variations in lesion development [10].

The symptoms of a defined experimental mastitis vary between different animals from mild to lethal. Interestingly, the severity of clinical response seems to be relatively characteristic and constant for a given animal, as shown by repeated infection studies [11]. The most prominent early response of the mammary gland is a dramatic increase in somatic cell numbers in milk occurring after infusion of *E. coli *[12] or its lipopolysaccharide [13] solely in the infected udder quarter. Moreover the infection provokes systemic effects as e.g. evidenced by fever, increased level of TNF-alpha in blood [14] or enhanced expression of several genes like haptoglobin in neighboring quarters [15]. In addition, a systemic reaction is evidenced by altered gene expression in distant organs as shown for liver after infusion of lipopolysaccharide into the mammary gland [16]. Surprisingly, experimental infection of a second healthy udder quarter twelve hours after the primary infection does not change the somatic cell count in this quarter, while the increase in somatic cell numbers in the first infected quarter continues [12,17].

These observations favor the concept, that infection with *E. coli *provokes two distinct types of response to the pathogen. The first comprises locally restricted reactions with acute symptoms and inflammation of the infected udder quarter. The second occurs in infected as well as in neighboring healthy udder quarters and furthermore influences even distant organs as shown for liver [16]. This systemic response may impair progression of subsequent infections of neighboring mammary quarters [12,17]. The interplay of both local and systemic reactions may finally determine the course of infection in an individual animal. The elucidation of the underlying mechanisms may help to improve mastitis resistance of livestock populations by selective breeding and reveal new ways to treat infections with *E. coli*. Here we present a transcriptome profiling approach to study the impact of an experimental *E. coli *infection on spatial gene expression profiles of the bovine mammary gland and to distinguish between local and systemic effects. The experimental system comprises dairy cows with defined lactation and immune status as hosts and *E. coli *1303 as pathogen. Microarray analyses of mammary gland tissue were performed in the early (6 h) and the late (24 h) phase of experimental infection. Udder tissue from healthy, untreated cows served as control for the discrimination between local and systemic effects in infected animals. We found, that infection of a single udder quarter with *E. coli *1303 induced substantial transcriptome changes both in the infected and in the neighboring udder quarters with more than two thousand differentially expressed genes. Cluster analyses of DEG revealed that two different reactions took place. The first was locally restricted to the infected quarter whereas the second occurred systemically in infected as well as in their neighboring noninfected quarters. Both reactions were characterized by gene ontology analyses and by searching of putative regulatory elements in the promoter regions of the DEG. Our results offer reasonable hypotheses why, after initial infection of one udder quarter, massive expansion of the bacteria in neighboring quarters is suppressed.

## Results

### Establishment of intramammary infection

**Figure 1:**
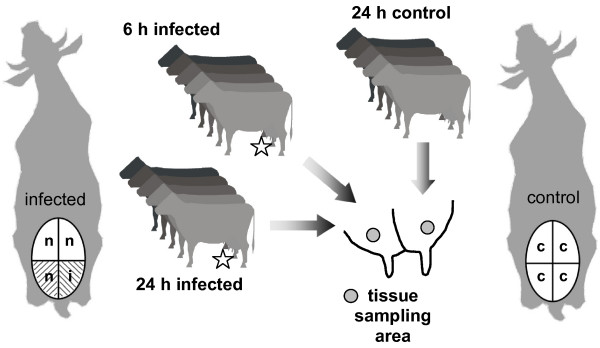
**Experimental setup of infection with *E. coli *1303**. During estrus, 10 synchronized dairy cows were infected on one quarter with *E. coli *1303 (star, i) in saline solution (shaded). One of the neighboring quarters (n) was treated with sterile saline solution (shaded). Five synchronized healthy animals were slaughtered 24 h after onset of estrus and served as external control (c). Tissue samples were collected from marked areas of the bovine udder.

**Figure 2:**
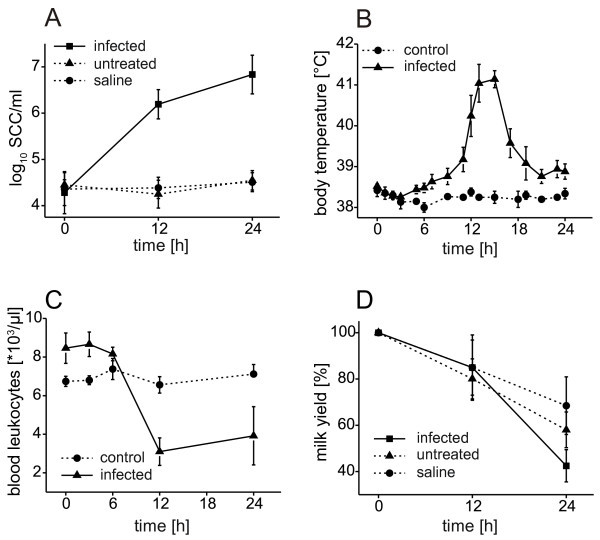
**Results of clinical and paraclinical findings**. Time course of A: somatic cell count in the milk of infected and neighboring noninfected udder quarters; B: rectal body temperature; and C: leukocyte counts in blood of infected and healthy control animals. D: milk yield of infected and neighboring noninfected (untreated or saline treated) udder quarters. The data shown are mean values and their corresponding standard errors and do not include values from animals of the 6 h infection group.

Prior to infection, the udder secretions of all animals had repeatedly been tested negative for major and minor mastitis pathogens. Following administration of 500 cfu of *E. coli *1303 to a single udder quarter and the same volume of physiological saline into a neighboring quarter of each of five cows (Figure 1), all inoculated animals suffered from acute mastitis and showed a strong cellular response. Major clinical symptoms, such as udder swelling and elevated counts of somatic cells in milk, were restricted to pathogen-inoculated udder quarters (Figure 2A). All milk samples from the inoculated quarters were positive for *E. coli *bacteria whereas neighboring quarters and all quarters of control animals remained bacteriologically negative during the observation period.

### Body temperature, milk yield, differential blood cell counts

All infected cows developed fever (>39.2°C) within the first 14 h after infection with maximal values (41.1 ± 0.2°C) between 15 h and 18 h after inoculation (Figure 2B). This was accompanied by significant leukopenia, reaching minimal blood leukocyte counts 12 h after pathogen inoculation (Figure 2C). Milk yield dropped progressively within the 24-h period after infection (Figure 2D), with a stronger decrease in infected quarters than in neighboring quarters. In infected quarters the number of somatic cells in milk increased starting between 6 and 12 h after infection (Figure 2A). In mock inoculated and untreated neighboring udder quarters of infected animals no significant increase in the number of somatic cells was observed.

### Microarray analysis

Mammary tissue samples were used to generate four sets of transcriptome profiles from: i) inoculated quarters 6 h post infection; ii) inoculated quarters 24 h post infection; iii) noninfected quarters neighboring the inoculated quarters 24 h post infection; and iv) quarters of untreated healthy control animals. Samples of sets iii and iv were generated by combining tissues of two udder quarters from each cow in order to equalize potential quarter specific effects. Based on somatic cell counts there was no indication for a different behavior of mock inoculated and untreated udder quarters of infected cows. Therefore tissues of two quarters with the lowest somatic cell counts were combined (see Additional File 1) provided their cell counts did not differ by more than 10.000 per ml. Otherwise tissue from the udder quarter with the lowest cell count was used. Thus four samples of set iii were pooled from one mock inoculated and one untreated udder quarter. The fifth sample (sample ID n24h-4) was derived from tissue of a single untreated quarter since the noninfected udder quarters of this cow differed in somatic cells counts.

**Figure 3:**
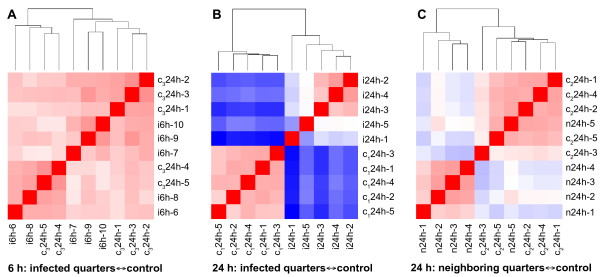
**Hierarchical clustering of transcriptome profiles after experimental infection with *E. coli***. Tissues of udder quarters of five healthy untreated cows (c24h-1 to 5) were used to generate three replicates of expression profiles as controls for three separate comparisons. A: Replicate 1 (c_1_24h-1 to 5) was compared to patterns of infected udder quarters 6 h after inoculation with *E. coli *1303 (i6h-6 to 10). B: Replicate 2 (c_2_24h-1 to 5) was compared to patterns of infected udder quarters 24 h after inoculation (i24h-1 to 5). C: Replicate 3 (c_3_24h-1 to 5) was compared to patterns of noninfected quarters of infected cows 24 h after infection (n24h-1 to 5). Samples for n24h-1, 2, 3 and 5 were pooled from one mock inoculated and one untreated udder quarter, while n24h-4 was derived from tissue of a single untreated quarter. The degree of similarity of their expression patterns is shown as a heatmap of the distance matrix (red: more similar, blue: less similar). The trees depict the pair wise relations with branch lengths proportional to the degree of similarity.

The impact of the *E. coli *infection on gene expression was estimated by hierarchical clustering of the transcriptome profiles (Figure 3). The heatmap obtained with the transcriptome profiles 6 h after inoculation showed a uniform coloration with no separation of healthy controls and infected samples (Figure 3A). This indicated that the gene expression of the mammary tissue was only marginally affected in the early stage of infection. The same comparison 24 h after infection with *E. coli *resulted in a sharp separation of the infected and the control group (Figure 3B) and a distinct blue-red coloration indicated substantial transcriptome changes. At the same time point, the effect of *E. coli *on transcriptome changes in neighboring noninfected udder quarters was less pronounced (Figure 3C). Compared with Figure 3B, the length of the vertical branch between the two principal groups is considerable lower, indicating a less pronounced difference of the expression profiles.

Nevertheless two almost perfectly separated groups were obtained: three of four noninfected samples pooled from one mock inoculated and one untreated udder quarter (n24h-1, -2, -3) clustered together with the sample derived from a single untreated quarter (n24-4), while the fourth pooled sample (n24h-5) was placed into the well-separated control group.

DEG (see Additional File 2) were identified by comparison of transcriptome profiles of infected and neighboring quarters with the profiles of the control animals. The highest number of DEG was found 24 h post infection in infected quarters (2154 at 0.01 fdr), followed by 476 (0.01 fdr) in their neighboring noninfected quarters and only 13 (0.1 fdr) in infected quarters at 6 h after inoculation. Despite the noisy data at 6 h after infection 6 of the DEG were also detected as differentially expressed at 24 h in infected quarters and 2 in neighboring quarters. At 24 h post infection, 294 DEG of the infected udder quarters were found among the DEG of the neighboring noninfected quarters.

This overlap indicates either the existence of a systemic response to a local infection with *E. coli *or a local effect after application of 2 ml PBS in mock inoculated and infected udder quarters. The first reaction should take place in all udder quarters of infected cows, the second however should be absent in their untreated ones.

### Local and systemic responses to *E. coli *revealed by cluster analysis

**Figure 4:**
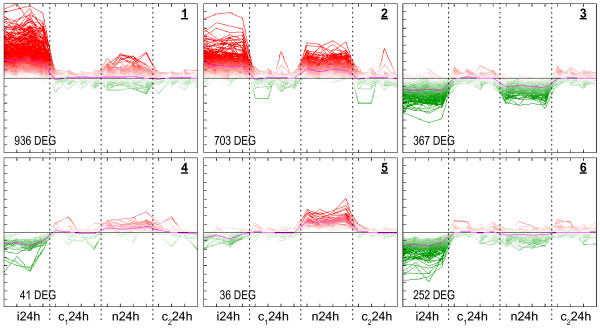
**Cluster analysis separates systemic and local response to infection**. Expression levels of DEG 24 h after *E. coli *infection were converted to log_2 _fold changes relative to the mean of untreated control animals. The four samples labeled with i24h represent i24h-1 to 4 of infected udder quarters. c_1_24h comprises the four samples c24h-1, 2, 4 and 5 derived from udder quarters of untreated controls. The four samples subscripted with n24h were isolated from udder quarters neighboring to the quarters i24h-1 to 4 and the four samples labeled with c_2_24h are replicates of c_1_24h. These technical replicates allow a common representation of the two separately performed analyses of i24h and n24h. Expression values were clustered using the SOTA algorithm with Pearson's correlation metric in the program MeV4.2. Individual probe sets are represented as lines, red coloration indicates up-regulation upon treatment, green down-regulation. Magenta represents the per sample average.

In order to look for local and systemic responses to *E. coli *infection, all DEG detected in infected quarters (2154) and noninfected neighboring quarters (476) were subjected to self organizing tree algorithm (SOTA) [18] clustering (Figure 4).

The calculated clusters revealed distinct reactions of the mammary gland in response to inoculation with *E. coli*. The first occurred exclusively in infected quarters (clusters 1 and 6) and comprised the local restricted response of the infected tissue. The second reaction took place both in infected and neighboring noninfected udder quarters (clusters 2 and 3) and pointed to a systemic response of the mammary gland to inoculation with *E. coli *in 2 ml PBS. Another type of reaction was recorded in the clusters 4 and 5. These 78 DGE were found induced specifically in the neighboring quarters, whereas in the infected quarters they were repressed (cluster 4) or unchanged (cluster 5). Their transcription was apparently regulated in a location specific manner. Nevertheless the different transcript levels in infected and neighboring noninfected udder quarters indicated that systemic signals are involved in their regulation.

The extent of apparently systemic gene regulation displayed in clusters 2 und 3 is much greater than the overlap of DEG from infected and neighboring quarters (294 genes). Roughly half of the 2335 genes fall into clusters that showed expression changes in infected as well as in neighboring quarters. This discrepancy is caused by the fact that DEG in infected quarters generally showed higher changes in expression than DEG in neighboring quarters. Due to the fold-change cut-off, genes with low fold-change are not assigned to the DEG but are nevertheless considered by SOTA clustering. Conversely, some genes differentially expressed in infected as well as in neighboring noninfected quarters were clustered by SOTA with the genes expressed locally in infected quarters (Figure 4, cluster 1), most likely because of their much higher induction in infected tissue than in tissue of neighboring quarters (e.g. *S100A9, S100A12, SAA3, SDS, CP*). For interpretation, those DEG, however, were treated as systemically reacting since the differential gene expression was regarded superior to clustering.

Based on annotations of the DEG more detailed analyses were performed. The genes of each cluster were analyzed in search of regulatory elements within their promoter region. Furthermore we identified biological processes involved in the systemic or local response of the mammary gland to the pathogen, which were represented by significantly more genes than expected by random sampling.

Both functional descriptions and transcription factor binding site (TFBS) enrichment analyses showed specific results for the members of the six clusters (Figure 4 and Table 1). Many results were specific for either local or systemic clusters and suggested that this dichotomy is not random but is biologically meaningful.

**Table 1:** Transcription factor binding site enrichment in clusters

Cluster	TFBS	Promoter range [kb]^a^	Gene hits	Z-score^b^	Fisher score^b^
1 (local, 936 DEG)	RELA	-5/5	244	20.45	7.97E-05
	NF-kappaB	-5/5	287	12.30	2.94E-04
	REL	-2/0	206	19.26	4.04E-04
	ELF5	-5/5	530	16.69	5.71E-04
	STAT1	-5/5	119	12.44	5.84E-04
	SRF	-5/5	48	12.10	8.09E-04
	SPIB	-2/2	502	11.23	2.09E-03
	Fos	-5/2	393	10.51	2.90E-03

2 (systemic, 703 DEG)	NFIL3	-2/2	100	12.72	4.53E-03
	TP53	-5/2	3	13.36	3.87E-02
	HLF	-2/2	127	13.06	4.23E-02

3 (systemic, 367 DEG)	FOXD1	-2/2	128	13.09	5.19E-07
	Foxa2	-5/2	158	12.46	2.75E-06
	Fos	-2/2	146	13.04	2.77E-05
	Pdx1	-2/0	156	23.12	3.63E-05
	FOXI1	-2/0	93	14.90	9.69E-05
	SRY	-5/2	192	18.15	1.55E-04
	PBX1	-2/0	29	11.26	1.97E-04
	REL	-2/0	92	10.49	2.57E-04
	Foxd3	-5/2	150	12.46	8.87E-04
	Sox5	-5/2	192	12.63	9.38E-04
	Nkx2-5	-2/2	188	23.31	9.98E-04
	Lhx3	-2/0	81	14.12	1.10E-03
	Prrx2	-2/2	174	20.48	1.91E-03
	NFIL3	-5/2	78	10.24	1.37E-04

4 (systemic, 41 DEG)	PBX1	-2/2	9	10.26	1.89E-03
	STAT1	-5/2	9	12.03	7.36E-03

5 (systemic, 36 DEG)	HNF4A	-2/0	7	10.21	4.44E-02
	NR2F1	-2/0	4	12.14	9.58E-02

6 (local, 252 DEG)	Hand1-Tcfe2a	-2/2	129	10.40	2.31E-07

^a ^range of the considered promoter region in the human ortholog upstream (-) or downstream of the transcription start site (TSS)

^b^ranking metrics for TFBS overrepresentation, Z-score accounts for frequency and Fisher-score for presence/absence of a given TFBS

### Characterization of the local response 24 h after infection

**Figure 5:**
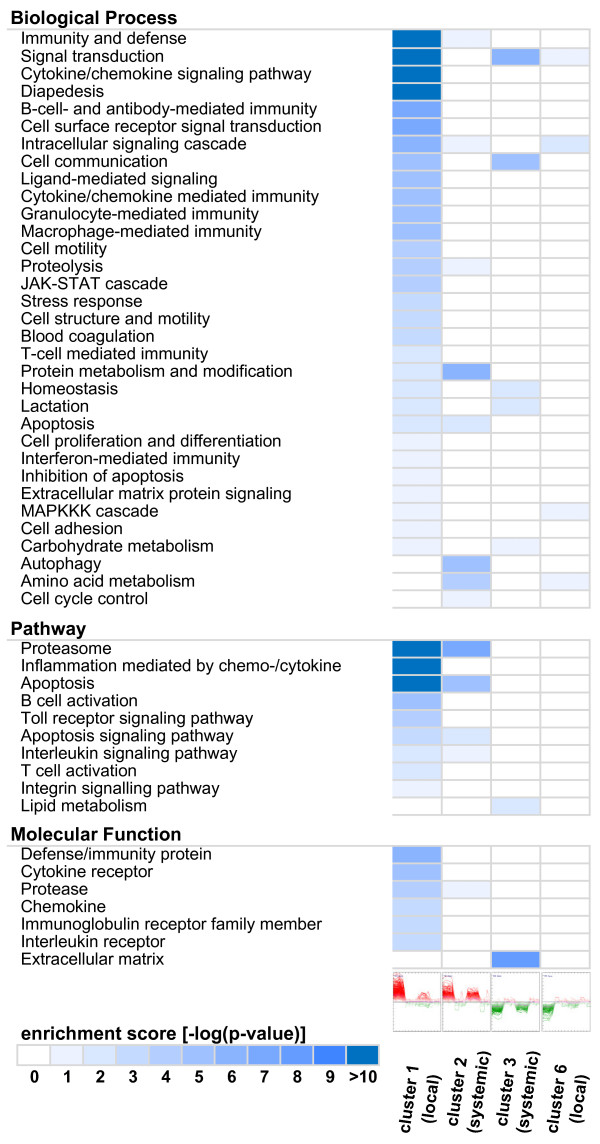
**Gene Ontology and pathway enrichment of clusters**. The genes constituting the clusters in Figure 4 were subjected to enrichment analysis using the Pantherdb gene expression tool and the CoPub keyword enrichment calculator. Biological processes, pathways and molecular functions that were significantly enriched (adjusted p-value < 0.05) in at least one cluster are shown as a heatmap. P-values are color coded ranging from white for non-significant results to blue colors saturating with increasing significance. Rows indicate the enriched terms and columns the p-values of the respective clusters. Clusters 4 and 5 did not yield any significant enrichment and were omitted.

Analysis of the enrichment of functional annotations revealed that the locally up-regulated genes of cluster 1 (Figure 4) have a distinct profile of functions that is not found among the members of the other clusters and that is dominated very prominently by ontology terms related to immune response and inflammation. In addition ontology terms like chemokine/cytokine signaling and diapedesis were among the most prominent. This correlated with our clinical findings of a sharp increase in leukocytes solely in the milk of infected udder quarters. Many genes of this cluster were associated with classical inflammatory events (Figure 5) and TFBS enrichment analysis (Table 1) predicted the classical inflammatory transcription factors NFκB and STAT1 as regulatory elements. Major genes related to fever induction, *IL6 *and *IL1B*, were still up-regulated (16-fold and 4-fold respectively) in infected udder quarters 24 h after infection although the inner body temperature was already declining and approached basal levels at this time point. Furthermore, the majority of observed acute phase genes (e.g. *HP*, *PTX3*, *FTL*) was assigned to cluster 1. As evidenced in Figure 5, almost all DEG of the ontology response to infection were found locally restricted to the infected quarters and only a few were observed also in neighboring quarters. The highly significant enrichment of biologically meaningful terms (e.g. adjusted p-value 1e-38 for 'immunity and defense') is also an important indication for the validity of the presented microarray data.

Besides the response to infection TFBS enrichment analysis gave hints towards other processes. Among the genes of cluster 1, ELF5 was predicted as involved transcription factor. ELF5 is related to development of mammary epithelium [19] and milk production [20].

### Characterization of the apparent systemic response 24 h after infection

The transcriptional response in the quarters neighboring the infected ones could in part be characterized as an immune response. Components of the antigen processing and presenting machinery as well as some cytokines are up-regulated. Of note are *CXCL2 *and *CXCL14*, whose products show direct antimicrobial properties against *E. coli *[21] and could probably function as a barrier against invasion of *E. coli *into the neighboring udder quarters. DEG of other important immune-related agents, as for example the CC-type of chemotactic cytokines, were restricted to the infected udder quarter. A number of genes, like *GPX3*, *GLRX2*, *GSTM1*, *GSS*, *MT1A, MT2A *and *SOD2*, indicate the presence of oxidative stress. Differential gene expression related to xenobiotic stress is evident by up-regulation of *ATF5*, which is responsible for the induction of the detoxifying cytochrome CYP2B6 [22], the latter being up-regulated only in the neighboring quarters (cluster 2).

Finally, there are signs of building up a first line of defense, like the up-regulation of genes with antimicrobial functions (*S100A8/9/12*, *GNLY*, *CXCL2*, *CXCL14*) or acute phase genes (*SAA3*) involved in recognition of pathogens (*LBP*) or in the complement cascade (*C6*, *BF*, *C4BPA*, *IF*).

### NFIL3 as a candidate for signal transduction

Among the predicted transcription factors for the DEG, NFIL3 was the most interesting candidate. Its binding motif was enriched in the clusters of both systemically up- and down-regulated DEG (clusters 2 and 3), in line with its function as activator/repressor of inflammatory and apoptotic genes. Furthermore, *NFIL3 *was found in infected quarters as 3.5-fold up-regulated DEG 24 h after infection. Interestingly, up-regulation of *NFIL3 *expression was found as soon as 6 h after infection. Five of the DEG at 6 h belong to the apparently systemically regulated genes at 24 h and only 3 of them were found local in infected udder quarters. Interestingly at 6 h the DEG also comprised a couple of predicted NFIL3 target genes, and TFBS for NFIL3 were significantly enriched. The putative target genes regulated by NFIL3 suggest a systemic up-regulation of genes involved in cell proliferation (e.g. *KLF6*, *FGF2*) as well as apoptosis and a down-regulation of genes related to cell adhesion and extracellular matrix.

### Analyses of mRNA expression by quantitative real-time RT-PCR

In order to verify the results of microarray analyses, we selected 14 genes as representatives of all SOTA-clusters (Figure 4) and used RT-qPCR to quantify their expression in infected udder quarters and in the neighboring saline treated or untreated quarters. The results were referenced against the expression in udder quarters of healthy animals. As summarized in Table 2 (Additional File 3), the values for induction and repression of transcription obtained by microarray analyses were clearly confirmed by quantitative PCR. In general the calculated fold-changes were higher for qPCR. For example, the expression of *SAA3 *was found to be 1825-fold induced in infected udder quarter 24 h after inoculation with *E. coli *1303, whereas microarray hybridization detected a 52-fold induction.

**Table 2:** Validation of microarray results by quantitative real-time RT-PCR (qPCR)

		Mean fold change qPCR				Mean fold change Affymetrix	
			
Gene	SOTA Cluster	i24h	Øn24h	n24h_u	n24h_s	i24h	n24h
SF3A1	-	-	-	-	-	-	-
HP	1	776	3.7*	3.5	4.3	207*	3.6
SAA3	1	1826*	5.3*	5.3	5.7	51.6*	5.4*
PTX3	1	32.7*	1.2	1.4	1.0	19.0*	1.2
S100A8	2	1351*	11.3*	13.9	9.2	165*	11.2*
MT2A	2	921*	63.7*	73.9	51.0	26.0*	58.8*
LBP	2	27.9*	4.0*	3.7	4.6	19.3*	3.8*
AQP3	3	-7.5*	-3.5*	-3.5	-3.5	-3.3*	-3.4*
ALOX15	3	-48.4*	-11.8*	-11.8	-11.8	-26.2*	-12.9*
ACAS2	4	-7.4*	1.2*	1.2	1.2	-2.3*	1.3*
ADHFE1	4	-2.6*	1.3	1.2	1.3	-2.1*	1.3*
PIR	5	-1.1	4.4*	5.3	3.2	-1.1	4.4*
THRSP	5	-3.3	3.8*	3.0	5.1	-1.8	3.6*
HPGD	6	-32.0*	-3.7	-3.5	-4.0	-12.3*	-3.7
LPL	6	-116*	-3.6*	-3.8	-3.2	-23.6*	-3.7

*: p Value < 0.01. Detailed data: see Additional File 3

In order to investigate the influence of the inoculation solution (2 ml of physiological saline), the expression of the selected 14 genes was determined in all sampled udder quarters of infected animals and the values of untreated udder quarters and mock inoculated udder quarters were compared. As shown in Additional File 4, the treatment of the neighboring udder quarters with saline did not provoke a significant difference in expression of these 14 genes.

The results of qPCR allowed an estimation of animal specific variation of gene expression. The ΔCP values summarized in Additional File 5 clearly demonstrate that the transcript levels of some genes like *HP *or *MT2A *differed up to one order of magnitude in infected udder quarters 24 h hours after inoculation with *E. coli *1303, whereas other genes like *PIR *or *LBP *were found to be uniformly expressed among the different animals.

## Discussion

In this work, the transcriptome response of the bovine mammary gland was analyzed after experimental infection with *E. coli *1303. Previous observations in cattle [10,12,23] favor the hypothesis of a systemic defense reaction, which protects unaffected udder quarters. In order to study this phenomenon, we investigated the transcriptome of bovine mammary tissue after infection with *E. coli *1303 in the early stage of infection (6 h) before onset of fever and in the late stage (24 h) after its decline. As described for *E. coli *1303 [17] infection of a single udder quarter induced acute clinical mastitis in all inoculated animals with comparable clinical signs such as strong fever, leukopenia, decreased milk yield and influx of somatic cells into the infected quarter. The somatic cell count (SCC) in mock inoculated as well as untreated udder quarters of the infected animals remained unchanged at all time points.

At 6 h after infection only 13 DEG were captured solely in infected quarters, indicating subtle effects of either the pathogen or the inoculation solution itself on the mammary gland at this time point. Furthermore we did not observe enhanced expression of proinflammatory cytokines like TNF-alpha. The missing induction of proinflammatory molecules in the early phase of infection and the unchanged SCC in mock inoculated udder quarters up to 24 h proves that the inoculation procedure neither provoked inflammation nor an increase of somatic cells in milk.

At 24 h after infection we observed marked changes in gene expression in neighboring quarters. These could be caused by a systemic response to the pathogen or a direct effect of the mock inoculation with 2 ml of PBS. As described by Paape et al. [24] the inoculation of even 100 ml saline solution provoked only a mild increase of SCC. Our procedure based on the application of 2 ml saline did not change the SCC of mock inoculated udder quarters as shown in Figure 2A. Furthermore the analysis of transcript levels of 14 selected genes by qPCR did not show significant differences between untreated and mock inoculated udder quarters (Additional File 4) and proved the existence of a systemic reaction. A detailed look on the setup of the microarray hybridizations (Additional File 1) reveals, that four profiles (n24h-1, 2, 3, 5) are based on pooling of samples from a mock inoculated and an untreated udder quarter, while the profile n24h-4 was obtained from a single untreated quarter. Assuming that the transcriptome changes observed in the neighboring quarters are predominantly caused by the saline treatment, the profile n24h-4 should cluster together with the controls c24h1-5. However, the highly similar expression profiles of the untreated sample n24h-4 and other neighboring samples (Figure 3C) show that the systemic effect of the pathogen by far dominates a possible effect of the saline treatment. The notion that 2 ml PBS has little influence is further substantiated by the assignment of profile n24h-5 to the cluster of the controls. Hence the saline treatment did not markedly alter the transcriptome of the pooled sample n24h-5 compared to the control group. Taken together the above observations strongly suggest that the experimental infection of an udder quarter caused an effect on the neighboring udder quarters, which we believe to be mediated systemically.

By comparison with expression profiles of healthy animals, large numbers of DEG were identified at 24 h in infected and a lesser number in neighboring noninfected quarters. Cluster analysis identified at least two categories of DEG. The first was found in infected quarters and comprised the local response of the infected udder quarter. DEG of the second category were found in inoculated and noninfected udder quarters of infected animals. This presumptive systemic response was discovered by comparison of noninfected neighboring quarters and samples of healthy animals. In contrast, most experimental designs used in mastitis research lack external controls and are thus not able to differentiate between local and systemic responses to the pathogen [12,17,25,26]. A substantial systemic transcriptome response in noninfected neighboring quarters was proposed [27], but the experimental outline precluded further characterization. Recently, systemic effects of microbial induced mastitis have been found in the bovine udder by analysis of composition and viability of somatic cells in milk [23] and in the liver transcriptome of intramammary LPS-challenged cows [16]. Comparison of the latter with our findings by gene set enrichment analyses resulted in a higher coincidence with the systemic response than with the local response of infected udder quarters (data not shown).

Cluster analyses revealed very similar expression profiles of all infected udder quarters and a pronounced response to infection, probably due to the pathogenicity of *E. coli *1303. In contrast, the expression profiles of noninfected udder quarters were more variable and one was rather similar to untreated animals (n24h-5 in Figure 3C). This observation clearly demonstrates that animal specific factors affected the systemic reaction, although the animals included in our study were selected according to strict criteria regarding the udder health.

The DEG were analyzed in the context of biological pathways, gene ontologies and predicted transcription factor binding sites to facilitate a better understanding of the local and the systemic reaction. Generally we found a clear division of biological functions between both types of reactions (Figure 5). DEG of the local reaction are mainly involved in immune response, inflammation, acute phase response and chemokine/cytokine signaling. TFBS analyses based on human orthologs predicted the corresponding transcription factors like NFκB and STAT1. As shown by Zadissa et al. [28], the use of promoter sequences from human genes for the inference of bovine transcriptional regulators is appropriate. The predicted factor ELF5 probably refers to DEG related to the restoration of damaged epithelial tissue, which is also supported by the enrichment of ontology terms like blood coagulation, cell proliferation and differentiation in cluster 1 (Figure 5). The majority of the DEG involved in immune response was assigned to the local reaction and only a few were also found in neighboring noninfected quarters. In this context, a detailed analysis of expression of chemokine genes led to a remarkable result. Enhanced expression of CC-type chemokine genes was exclusively assigned to the local reaction, whereas many CXC chemokine genes were regulated systemically. In contrast to the CC-type, CXC chemokines are known to attract neutrophils. The enhanced systemic expression of genes involved in response to oxidative stress accounts for the influx of active neutrophils in all quarters. This corresponds with the finding, that acute microbial mastitis caused a significant increase of the percentage of neutrophils in neighboring unaffected quarters [23]. The systemic reaction comprises biological processes like antigen processing and presentation, protein degradation, apoptosis, autophagy and genes of cytokines. Compared with the local reaction it includes only parts of an immune reaction and could be considered as well balanced conditioning process facilitating the progression of the immune response. This is for example illustrated by the induction of genes related to apoptosis, which was described to counteract adherence of bacteria in epithelia by exfoliation of the outermost layers [29]. The early onset of this defense reaction is indicated by enhanced expression of *NFIL3 *already 6 h after infection. In the late stage of infection, NFIL3 was predicted as regulatory element of many DEG involved in the local as well as the systemic reaction. Thus, the NFIL3 signal might promote a remodeling of the epithelium in both, infected and adjacent udder quarters. Studies in mice suggested that *Nfil3 *is involved in the functional regulation of the mammary epithelium [30]. Altered epithelial cell functions might cause the decrease in milk production observed in our study in all quarters (Figure 2D). Furthermore the ontologies 'lactation' and 'lipid metabolism' were prominent among systemically down-regulated DEG (cluster 3). This correlates with the finding that infection with *E. coli *caused reduction in milk yield in infected as well as in noninfected neighboring udder quarters (Figure 2D). Interestingly, at 24 h after infection we observed enhanced systemic expression of antiapoptotic genes (e.g. *CFLAR*, *MCL1*). This may be part of a counterbalance reaction to normalize the function of the mammary epithelium after the observed decline of fever.

Our results favor the hypothesis that very early after contact with *E. coli *signals are transmitted to the surrounding tissue and to the neighboring quarters. They induce a systemic reaction in all udder quarters that apparently prevents multiplication of the pathogen in neighboring quarters and influences the progression in the infected quarter. It explains, why time-shifted inoculations of different udder quarters with the same (low) pathogen titer caused influx of leukocytes only in the udder quarter first infected [12,17]. Our hypothesis is also in line with the phenomenon of recurrent mastitis in different udder quarters caused by the same environmental strain of *E. coli *[10]. The pathogen may be transferred from the first infected udder quarter to neighboring quarters, but the ongoing systemic reaction inhibits multiplication of the pathogen in the time delayed infected quarters.

**Figure 6:**
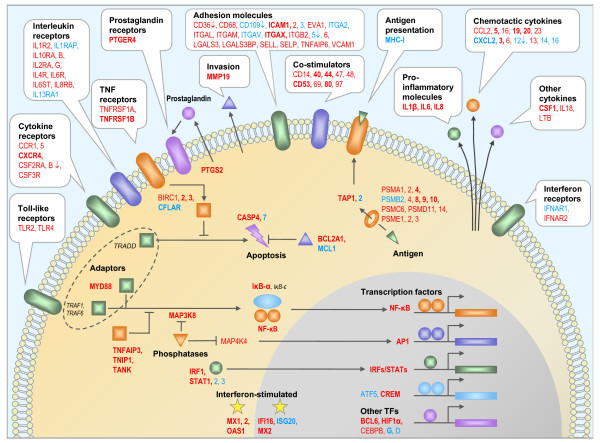
**Local and systemic immune response of the bovine udder 24 h after infection**. A graphical representation of a robust response to infection as detected by a series of microarray experiments was adapted from [31]. Genes that were differentially expressed in the bovine udder upon *E. coli *infection are listed, further differentiating between systemic reactions in blue and local reactions in red. Bold letters indicate accordance with [31]. Genes were up-regulated unless marked with an arrow, indicating that expression is repressed upon infection.

By integration of our findings into a consensus scheme of the transcriptional response to infections [31] we could provide a detailed picture of the processes occurring in the bovine mammary gland 24 h after inoculation with *E. coli *1303 (Figure 6) and add a new functional dimension by differentiating locally and systemically regulated genes. The latter group points to altered milk composition and synthesis of protection factors, such as antimicrobials and reactive oxygen species in the late stage of infection. The systemic reaction could effectively prevent growth of *E. coli *and formation of clinical signs in neighboring udder quarters and could thereby moderate neutrophil influx. The significance of our results is evidenced by some recent findings with candidate gene based approaches. We found that *TLR2 *was 6-fold up-regulated 24 h after infection solely in infected quarters and accordingly *TLR4 *2-fold. Enhanced expression of *TLR2 *and *TLR4 *was measured by RT-PCR in the pathogen inoculated udder quarters and furthermore expression of TLR2 was detected by immunohistochemistry in mammary epithelial cells [17]. Milk of infected animals was described to contain elevated levels of the acute phase proteins haptoglobin (HP), serum amyloid A (SAA), lipopolysaccharide binding protein (LBP) [32] and alpha-1-antitrypsin (SERPINA1) [33]. We found *HP *200-fold up-regulated locally in infected quarters, while *SAA3*, *LBP *and *SERPINA1 *showed systemically increased expression in infected (*SAA3*: 60-fold; *LBP*: 20-fold; *SERPINA1*: 10-fold) as well as in neighboring uninfected quarters (*SAA3*: 3-fold; *LBP*: 4-fold; *SERPINA1*: 3-fold). S100 calcium-binding protein A12 (S100A12) which was shown to have a direct antimicrobial effect on *E. coli *[34] was also identified in milk of cows with *E. coli *induced mastitis [33]. In our study *S100A12 *was up-regulated in the infected (110-fold) as well as in the neighboring quarters (4-fold). Previously high levels of interleukin 8 were measured in milk of infected cows [35]. We found 120-fold enhanced expression of *IL8 *in infected udder quarters 24 h after infection and no significant change in neighboring quarters. IL8 was shown to activate and attract neutrophils [36]. The observed enhanced expression of *IL8 *in infected quarters might be involved in the dramatic influx of somatic cells observed in these quarters only (Figure 2A). The increase of SSC was described to correlate with enhanced expression of lingual antimicrobial peptide (LAP) and its expression in epithelium was confirmed by in situ hybridization solely in the infected udder quarter [37]. At 24 h after infection, we found a 20-fold induction of *LAP *solely in infected quarters. These examples demonstrate the correlation of our findings with results of previously published candidate gene based investigations of bovine mastitis.

## Conclusion

In summary, this first comprehensive description of complex systemic effects in the mammary gland after local *E. coli *infection introduces a new concept to mastitis research and may yield new targets for prevention and therapy of mastitis. Moreover, the observed variation in the systemic response between individual animals could be the basis for selective breeding towards resistance against *E. coli *induced mastitis.

## Methods

### Animals

This study included 15 healthy German Holstein Frisian heifers in mid lactation (3 to 6 months post partum). The trials were conducted at the Clinic for Ruminants, LMU Munich (Oberschleißheim, Germany) with the approval of the ethics committee of the regional government of Upper Bavaria, Germany (No. 55.2-1-54-2531-108-05). Only animals without previous diagnosis of clinical or subclinical mastitis and a reported somatic cell count <50,000/ml were included in the study. Quarter milk samples were collected and tested weekly before the trial to ensure that they contained <50,000 somatic cells/ml and were free of mastitis pathogens. Two different infection models were used in which the animals were inoculated in one quarter with *E. coli *and killed after 6 h (n = 5) or 24 h (n = 5), respectively. Five heifers served as controls, received no treatment and were killed after 24 h.

### Somatic cell count (SCC) and bacteriological examination

Three weeks before the trial the following parameters were documented twice daily for each animal: milk yield, somatic cell count (SCC, California Mastitis Test, CMT) and rectal body temperature. Quarter milk samples were collected weekly for determination of the SCC and bacteriological examination. The SCC was determined with a Fossomatic 5000^® ^(FOSS Electric, Hillerod, Denmark) cell counter. To check for the absence of bacteria, 15 μl quarter milk samples were plated on Columbia sheep blood agar, Gassner agar and Edwards agar (Oxoid, Wesel, Germany). Samples were incubated for 48 h at 37°C.

### Inoculum dose

Bovine mastitis isolate *E. coli *1303 belongs to the major *E. coli *phylogenetic group A (*E. coli *collection of reference strains, ECOR-A). According to a multiplex PCR-based screening for virulence-associated genes of pathogenic *E. coli*, this strain does not represent an extraintestinal or intestinal pathogenic *E. coli *isolate. Only the genes coding for type 1 fimbriae, F17 fimbriae, antigen 43, the ferric citrate siderophore system and the EAST1 toxin could be detected. Bacteria were kept cryo-conserved (Mikrobank-System Cryobank™, Mast Diagnostika, Reinfeld, Germany) for subsequent infections. Bacteria were plated on Columbia sheep blood agar and incubated (37°C) for 24 h. A few colonies were transferred to a tube of brain-heart infusion broth (Oxoid, Wesel, Germany) and incubated for 6 h (37°C); then a 100-μl sample was transferred to a tube of trypticase soy broth (9.9 ml, Oxoid, Wesel, Germany). Serial dilutions were made after 18 h to prepare the desired inoculum dose of 500 cfu/2 ml 0.9% sterile, pyrogen-free saline. The inoculum dose was plated for control and ranged from 421-613 cfu/2 ml.

### Experimental pathogen inoculation, sampling of milk, blood, and tissue

To achieve comparable hormonal conditions, cows were synchronized by two injections of (+)-cloprostenol (Dalmazin^®^, Selectavet, Weyarn, Germany) at 12-day intervals. Animals were challenged three days after the second injection during estrus. 20 IU oxytocin (Veyx-Pharma, Schwarzenborn, Germany) were applied intravenously and the udder was entirely milked out. Teats were cleaned and disinfected with 70% ethanol and 500 cfu *E. coli *strain 1303 were administered intracisternally in one quarter through the teat canal. Two milliliters of 0.9% sterile pyrogen-free saline without bacteria were inoculated into another quarter as placebo. The control group was treated the same way but without inoculation of an udder quarter.

The udder secretions were sampled in 12 h intervals before regular milking. Bacteriological examination was performed from foremilk samples, the SCC was determined in 10 ml whole milk after each udder quarter had been milked separately with a quarter milker (WestfaliaSurge, Bönen, Germany).

Blood samples (10 ml) were taken from the jugular vein aseptically using EDTA-vacutainers (Becton Dickinson, Heidelberg, Germany) at 0, 3, 6, 12 and 24 h after start of the trial. Finally, cows were killed 6 h or 24 h after trial start with a penetrating captive bolt gun followed by exsanguination. Leukocyte counts in blood samples were carried out with a Sysmex pocH100i (Sysmex, Norderstedt, Germany). Tissue samples were collected aseptically from slaughtered cows within 10 min after killing. A piece of tissue (5 × 5 × 5 cm) was removed from a deeper location of the udder quarter, 7 cm dorsal of the milk cistern, of which a smaller tissue piece (1 × 1 × 0.5 cm) was transferred to a tube containing 5 ml RNAlater^® ^(Applied Biosystems Incorporation, Foster City, USA) and served as original material for all further analyses.

### Microarray hybridization

Total RNA was isolated by disrupting small pieces of approx. 500 mg of bovine mammary gland in 5 ml of TRIzol reagent (Invitrogen, Carlsbad, USA) with a tissue homogenizer (Heidolph, Schwabach, Germany). Homogenate was processed following the TRIzol manufacturer's protocol to yield highly pure total RNA. Purity (A260/A280 >1.9, A260/A230 >2.2) and integrity of RNA (28S:18S rRNA ratio ~1.5:1) was assessed by spectrophotometry (Nanodrop ND-100, NanoDrop Technologies LLC, Wilmington, DE, USA) and agarose gel electrophoresis.

Labeled cRNA probes for array hybridization of the 24 h samples (infected udder quarters, neighboring quarters and controls) were prepared with an Ambion Kit (MessageAmp™ II-Biotin Enhanced Single Round aRNA Amplification Kit). The probes for analyses of the 6 h time point (infected udder quarters and controls) were prepared with an Affymetrix kit (GeneChip^® ^Expression 3' Amplification One-Cycle Target Labeling Kit). RNA samples from noninfected udder quarters of the same animal were pooled when they had comparable somatic cells counts (detailed description of samples: see Additional File 1). The controls were performed as replicates to ensure comparability of the sets.

10 μg of the resulting fragmented cRNA were hybridized overnight to GeneChip^® ^Bovine Genome Arrays (Affymetrix, Santa Clara, CA, USA) processed in an Affymetrix Fluidics Station 450 and scanned with the Affymetrix 3000 7G scanner. Hybridizations were performed in three sets of 10 arrays each: (set 1) 5 infected quarters 24 h after infection and 5 controls; (set 2) 5 samples from neighboring quarters 24 h after infection (see Additional File 1) and 5 controls; (set 3) 5 challenged quarters 6 h after infection and 5 samples from untreated controls. The statistic software R and packages from the bioconductor [38] microarray suite (affy, affyPLM) were used for processing the CEL files and to identify possible outliers due to technical or handling artifacts. The microarray data has been deposited in the Gene Expression Omnibus database (accession GSE15025).

### Statistical analysis and bioinformatics

Microarray raw data were summarized and normalized with the RMA method [39] and filtered for a detection criterion (MAS5 'present'-calls determined by Bioconductor's affy-package in R). DEG were identified by the packages LIMMA [40] and SAM [41]. LIMMA was performed for the analysis of the sample sets 24 h after infection, excluding profiles of animal 5 (samples i24h-5 and n24h-5) as a biological outlier and control animal 3 (sample c_1_24h-3 and c_2_24h-3) as a technical outlier. Due to its higher sensitivity in case of noisy data SAM was used for analysis of the 6 h time point. Cut-off criteria were a higher than 2-fold change and a false discovery rate of <1% for LIMMA and <10% for SAM analysis. The expression values of set 1 and set 2 for 2335 DEG were subjected to cluster analysis using the SOTA module of MeV4.2 [42] with the Pearson correlation as clustering metric and default values except for Winning Cell Migration Weight (0.006). The number of clusters was set to six, based on figure-of-merit calculations. The members of the resulting clusters were subjected to enrichment analyses with different online tools e.g. pantherdb [43] and CoPub [44] for GO terms and pathways and oPOSSUM [45] for TFBS. These analyses were based on human orthologs of the DEG as described by Hintermair [46] and the human genome or the orthologs present on the microarray as the background. Enriched functional annotations and pathways are represented as a heatmap of the Bonferroni-adjusted p-values with a cut-off of 0.05. The human ortholog gene symbols of differentially expressed bovine genes are listed in Additional File 2.

### Real-Time RT-PCR

Quantitative real-time PCR reactions were performed with the same RNA samples as used for microarray hybridization using the LightCycler DNA Master SYBR Green I protocol [47]. The following primers were designed to amplify specific fragments referring to selected differentially expressed genes of each cluster (length of the PCR products in square brackets): cluster 1: *HP *(haptoglobin; for 5'-ACCTGGTATGCGGCCGGGA; rev 5'-TCCGAACCCAGTCCAGAATGGAGG [100 bp]), *SAA3 *(serum amyloid A3; for 5'-ATGACGCTGCCCGAAGGGGA; rev 5'-TGTCAGGCAGGCCAGCAGGT [210 bp]), *PTX3 *(pentraxin 3; for 5'-AAAGGGAGACTGGAGAAGGC; rev 5'-TGGCCAAAATGAAATTAACCA [173 bp]), cluster 2: *S100A8 *(S100 calcium binding protein A8; for 5'-AAAAAGGGAATTACCACGCC; rev 5'-ATCACCAGCACGAGGAACTC [163 bp]), *MT2A *(metallothionein 2A; for 5'-TCTCCGGACCCCAGCCTCCA; rev 5'-GCAGGAGGCGCACTTGCAATC [119 bp]), *LBP *(lipopolysaccharide binding protein; for 5'-CCTTGCCCTCAAACTCTCAG; rev 5'-CTTCCCCCTCCCTCTGTTAC [114 bp]), cluster 3: *AQP3 *(aquaporin 3; for 5'-TCGGTGGAGTTGGGTGGGGG; rev 5'-AGCCCCCTGAATAGAAGAAAGGGC [158 bp]), *ALOX15 *(arachidonate 15-lipoxygenase; for 5'-GGGTGGGATTCACCACGTGTCC; rev 5'-ACTGAGGCCAGATACCTCCAAC [170 bp]), cluster 4: *ACAS2 *(acyl-CoA synthetase short-chain family member 2; for 5'-GGCATGCACTTGCCCCGAGA; rev 5'-ACAGGCACTGCCATCCGGT [122 bp]), *ADHFE1 *(alcohol dehydrogenase iron containing 1; for 5'-AGGGCAGCCTGGACAAACGC; rev 5'-GATCCATGGAGCAAGGGCAGTC [102 bp]), cluster 5: *PIR *(pirin; for 5'-TTGGACCTGATGATGCACAGCAAA; rev 5'-ACGTGGACACTGTCACCTTCTCCC [76 bp]), *THRSP *(thyroid hormone responsive; for 5'-AAGTCAAAAGCATCTGGCATGT; rev 5'-ACTCAGAGTTGAGGACTCGGCTT [134 bp]), cluster 6: *HPGD *(hydroxyprostaglandin dehydrogenase; for 5'-TCTGTCTTCCACTGTAATGCTCAAAGC; rev 5'-AGTGAAGGCCACCAGTACAAAGACT [80 bp]), *LPL *(lipoprotein lipase; for 5'-TGACTTGTTGTTGGCATCCCCC; rev 5'-AAGTCAGAGTTCCCAGGGCCA [129 bp]) and *SF3A1 *(splicing factor 3a, subunit 1; for 5'-ACAAGGGTCCAGTGTCCATC; rev 5'-AGACCAGCACCTGTCCATTC [84 bp])) as housekeeping gene [48]. All amplified PCR fragments were sequenced (3100-Avant Genetic Analyzer; Applied Biosystems) to verify the resulting PCR product. In addition, the specific melting point (MP) of the amplified product carried out within the LightCycler standard PCR protocol confirmed product identity [49]. In each PCR reaction cDNA according to 50 ng total RNA was amplified in a total reaction volume of 20 μl (5 μM primer forward and reverse each, 1x LightCycler DNA Master SYBR Green I; Roche) using the LightCycler480 II instrument (Roche). The annealing temperature was 60°C for all PCRs.

### Data analysis of real-time RT-PCR

The cycle number required to achieve a definite SYBR Green fluorescence signal (= crossing point; CP) was calculated by the second derivative maximum method (LightCycler software, version 1.5.0). The CP is correlated inversely with the logarithm of the initial template concentration. The CP of the housekeeping gene *SF3A1 *showed no significant statistical difference in all analyzed samples. Therefore, it was used to normalize the CP for the target genes (ΔCP). Differences between values obtained for udder quarters of the control group and infected or neighboring quarters were stated by the ΔΔCP as well as the 'mean fold changes qPCR' [50]. The normal distribution was tested by the Kolmogorow-Smirnov method, followed by a Student's *t*-test to confirm significant differences between groups.

## Abbreviations

c: udder quarter of healthy cows; cfu: colony forming units; CP: crossing point; cRNA: complementary ribonucleic acid; DEG: probe sets of differentially expressed genes; *E. coli*: *Escherichia coli*; fdr: false discovery rate; GO: gene ontology; i: udder quarter inoculated with *E. coli*; LIMMA: linear models for microarray analysis; LPS: lipopolysaccharide; MAS: microarray suite; n: udder quarter neighboring to *E. coli-*infected udder quarter; qPCR: quantitative polymerase chain reaction; RMA: robust multi-array analysis; SAM: significance analysis of microarray data; SCC: somatic cell count; SOTA: self organizing tree algorithm; TFBS: transcription factor binding site.

## Authors' contributions

SM carried out the sample preparation, performed microarray analysis and qPCR, participated in bioinformatic analysis and contributed to writing of the manuscript. WP conducted the animal preparations and experimental infections, performed the evaluation of the clinical data and participated in writing the manuscript. SK performed bioinformatic and statistical analysis and contributed to drafting the manuscript. DM assisted in animal preparation and animal experiments. AK performed hybridization of microarrays. EW participated in the design of the study and performed writing and critical reading of the paper. HZ contributed to project conception and coordinated the animal experiments. HB conceived of the study, participated in interpretation of the results and drafted the manuscript. All authors read and approved the final manuscript.

## Supplementary Material

Additional file 1 Setup of microarray hybridizations and qPCR. Click here for file

Additional file 2 **List of differentially expressed genes**. List of differentially expressed genes Description Human ortholog gene symbols of differentially expressed bovine genes were used in the Additional File 2 and are based on unpublished data from Hintermair [46]. Click here for file

Additional file 3 **Detailed data of validation of microarray results by quantitative real-time RT-PCR (qPCR)**. CP, crossing point; ΔCP, CP-CP_SF3A1_; ΔΔCP, ΔCP_control _- ΔCP_treatment_; Øn24h, average of untreated (n24h_u) and saline treated neighboring quarters (n24h_s) of 24 h infected quarters. Click here for file

Additional file 4 **Expression profiles of 14 selected genes measured by real time RT-PCR**. The mRNA expression of *HP*, *SAA3*, *PTX3*, *S100A8*, *MT2A*, *LBP*, *AQP3*, *ALOX15*, *ACAS2*, *ADHFE1*, *PIR*, *THRSP*, *HPGD *and *LPL *were detected using real time RT-PCR (qPCR). The height of the bars indicates the average fold changes for the i24h and n24h group - the latter separated in untreated (n24h_u) and saline treated (n24h_s) -relative to the mean expression of the control. The error bars indicate the standard error of mean. Click here for file

Additional file 5 **Expression values of 14 selected genes in individual udder quarters measured by real time RT-PCR**. The mRNA expression of *HP*, *SAA3*, *PTX3*, *S100A8*, *MT2A*, *LBP*, *AQP3*, *ALOX15*, *ACAS2*, *ADHFE1*, *PIR*, *THRSP*, *HPGD *and *LPL *were detected using real time RT-PCR (qPCR). The samples **i24h-1 **to **5 **were derived from infected udder quarters of five animals 24 h after infection with *E. coli *1303. The five samples subscripted with **n24h1-5 **were isolated from udder quarters neighboring to the infected quarters and were untreated (**u**) or mock inoculated with 2 ml PBS. The samples labeled with **c24h **were taken from two udder quarters of 5 untreated control animals. The listed CP values represent the expression of each mRNA in relation to *SF3A1*. Click here for file
