# Supplementary figures and images for: Escherichia coli infection induces distinct local and systemic transcriptome responses in the mammary gland
Source: BMC Genomics. 2010 Feb 25;11:138. doi: 10.1186/1471-2164-11-138 (PMC2846913; doi:10.1186/1471-2164-11-138)

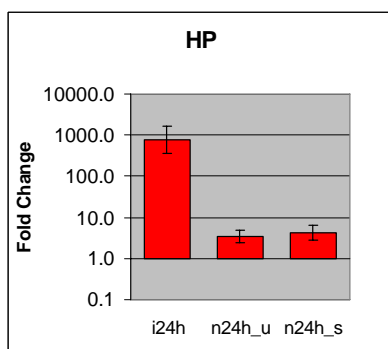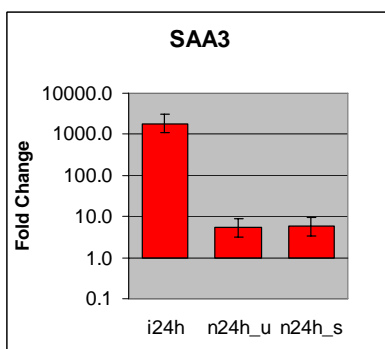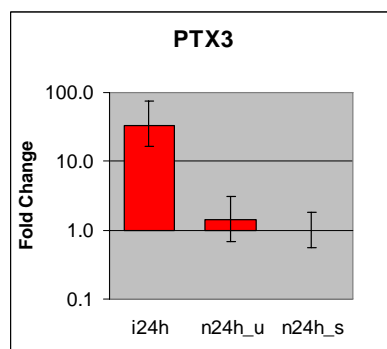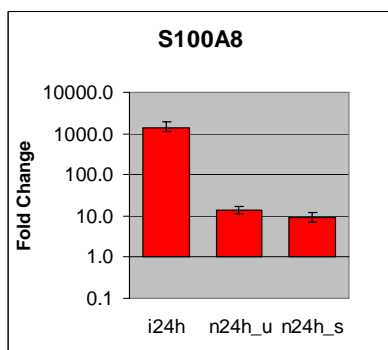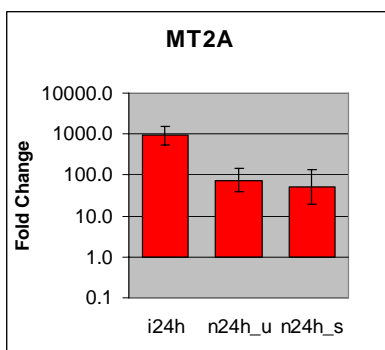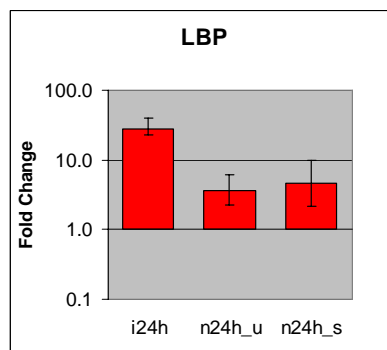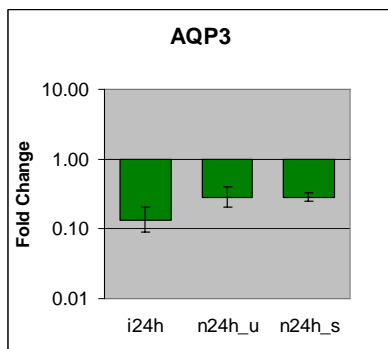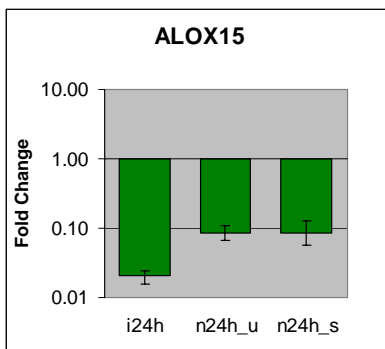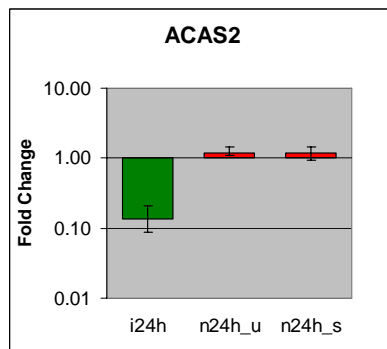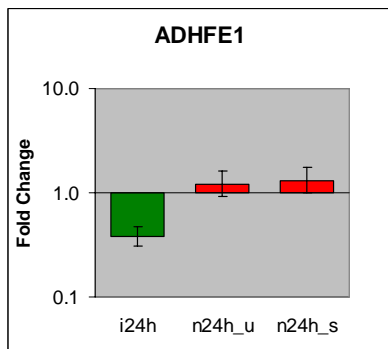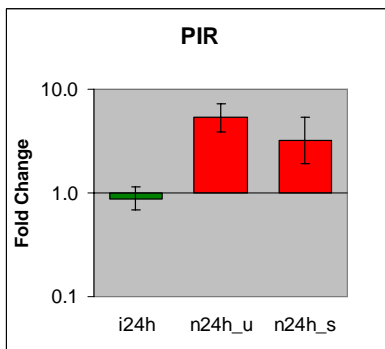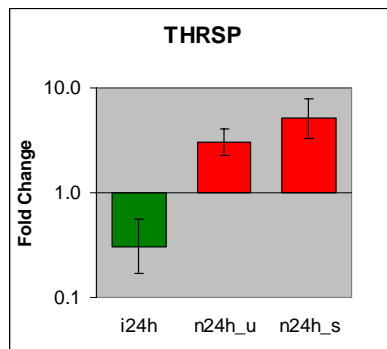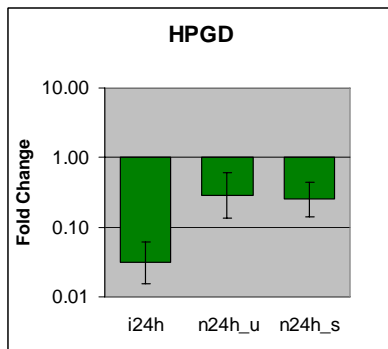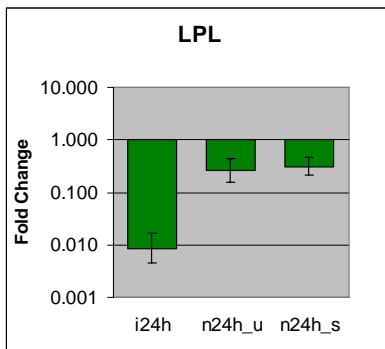

Supplement: Additional file 4 — Expression profiles of 14 selected genes measured by real time RT-PCR. The mRNA expression of HP, SAA3, PTX3, S100A8, MT2A, LBP, AQP3, ALOX15, ACAS2, ADHFE1, PIR, THRSP, HPGD and LPL were detected using real time RT-PCR (qPCR). The height of the bars indicates the average fold changes for the i24h and n24h group - the latter separated in untreated (n24h_u) and saline treated (n24h_s) -relative to the mean expression of the control. The error bars indicate the standard error of mean. [file 1471-2164-11-138-S4.PDF]
